# Weight Stigma and Avoidance of Physical Activity and Sport: Development of a Scale and Establishment of Correlates

**DOI:** 10.3390/ijerph192316370

**Published:** 2022-12-06

**Authors:** Nadia Bevan, Kerry S. O’Brien, Janet D. Latner, Chung-Ying Lin, Brian Vandenberg, Ruth Jeanes, Xavier C. C. Fung

**Affiliations:** 1 School of Social Sciences, Faculty of Arts, Monash University, Melbourne 3800, Australia; 2 Department of Psychology, University of Hawaii, Honolulu, HI 96822, USA; 3 Institute of Allied Health Sciences, College of Medicine, National Cheng Kung University, Tainan 701401, Taiwan; 4 Department of Occupational Therapy, College of Medicine, National Cheng Kung University, No. 1, University Rd., East Dist., Tainan 701401, Taiwan; 5 Department of Public Health, College of Medicine, National Cheng Kung University, No. 1, University Rd., East Dist., Tainan 701401, Taiwan; 6 Biostatistics Consulting Center, National Cheng Kung University Hospital, College of Medicine, National Cheng Kung University, No. 1, University Rd., East Dist., Tainan 701401, Taiwan; 7 Faculty of Education, Monash University, Melbourne 3199, Australia; 8 Department of Rehabilitation Sciences, Faculty of Health and Social Sciences, The Hong Kong Polytechnic University, Hung Hom, Hong Kong

**Keywords:** physical activity, avoidance, participation, scale development, weight stigma

## Abstract

Participation in sport and physical activity (PA) is declining, and the psychosocial factors underpinning avoidance of these activities are not understood. This study developed and tested a new measure assessing the tendency to avoid PA and sport because of weight stigma and appearance-related concerns. University students (*n* = 581, mean age = 19.8 years) completed an online survey at two time points. Demographic details and measures of weight stigmatization, appearance evaluations, and enjoyment and participation in PA or sport were taken. In addition, we developed and tested a new measure of the tendency to avoid physical activity and sport (TAPAS). Psychometric testing of the scale was conducted, and correlates of TAPAS were examined. The ten-item TAPAS provided a single factor solution, and the final scale score was predictive of lower levels of enjoyment of, and participation in, physical activity and sport (*p* < 0.001). The scale also displayed good internal and test-retest reliability. This study provides a new measure for assessing people’s tendency to avoid PA and sport because of weight stigma or appearance-related concerns. The results suggest that initiatives seeking to increase participation in PA and sport may need to address weight stigma and associated appearance related concerns.

## 1. Introduction

Declining participation in physical activity (PA) and sport worldwide is of concern to public health experts because of its importance in maintaining good health and reducing the costs of sedentary behaviour [1,2]. Research seeking to identify the potential barriers for greater participation in PA and sport has primarily focused on economic and environmental factors (e.g., cost, access, availability) [3,4]. However, efforts to address these structural factors have not halted the declining participation rates, which suggests other psychosocial factors (e.g., stigma, physical appearance concerns) may also influence individuals’ decisions to avoid participating in PA and sport.

Some research examining the relationship between experiences of weight stigmatisation, physical appearance concerns, and avoidance of PA and sport note the potential role of these psychosocial factors in PA and sport participation [5,6]. For example, experiences of weight stigma have been found to be associated with less motivation to exercise and the avoidance of exercise [7,8]. Similarly, physical appearance-related concerns such as body dissatisfaction [9] and fear of negative appearance evaluation [10] appear to play a role in the avoidance of PA and sport. In addition, some research on social physique anxiety suggests that anxiety around one’s body and appearance is related to the avoidance of PA and sport [11,12]. However, an examination of the concept of social physique anxiety, along with the measures used to assess it, suggests that this concept and associated measures are capturing body-image related dimensions rather than any distinct or new construct [11]. Women may be particularly likely to internalize sociocultural messages about ideal body shape and weight and, in turn, may avoid participating in activities such as PA and sport where their physical appearance may be judged [13,14]. It is noteworthy that women do have significantly lower levels of PA and sport participation compared to men [15], which may be due to the increased weight stigma and appearance-related concerns observed in women [16,17]. However, there is a paucity of research on the relationship between the avoidance of PA and sport and experiences of weight stigma and appearance-related concerns. This may be due to the absence of psychometrically sound measures designed to examine the tendency to avoid PA and sport because of appearance and weight-related concerns [6].

Although an adapted measure has previously been used to assess exercise avoidance motivation [18], this measure was ad-hoc in design and lacked psychometric testing. Other studies examining the relationship between exercise avoidance and body dissatisfaction have typically used one or two ad-hoc items because of the absence of a formal measure [e.g., 9]. In addition, the measures previously used have not directly examined the role of weight and appearance-related concerns in the avoidance of these activities. Appearance and weight-related concerns including weight stigma may also have a detrimental impact on the enjoyment of PA and sport, which also likely contributes to avoidance [5].

This study is part of a larger study identifying relationships between weight stigma, appearance-related concerns, and the avoidance of PA and sport in men and women [5]. The present study sought to develop and test a measure that could assess people’s tendency to avoid participating in PA and sport. Such measures could help researchers and policy makers to identify and address the psychosocial barriers that underpin the avoidance of PA and sport.

## 2. Materials and Methods

### 2.1. Participants

Six hundred and forty-seven undergraduate students were invited to participate in the study, which collected survey data from participants at two points, two weeks apart. The majority (89.7%; *n* = 581) agreed to participate and provided responses to the survey. Mean (M) age of the sample was 19.8 years (range 17–35 years), with a standard deviation (SD) of 2.3 years. The majority of the sample were women (*n* = 428; 73.8%). Participant Body Mass Index (kg/m^2^) was calculated from self-reported weights and heights (M = 22.47; SD = 3.44). Approximately 10.2% of respondents could be categorised as ‘underweight’ by BMI (BMI < 18.5), 71.5% as ‘normal range’ (BMI = 18.5–24.9), 14.3% as ‘overweight’ (BMI = 25–29.9), and 3.5% as ‘obese’ (BMI ≥ 30) [19]. At time one, five students did not provide a response regarding sex, height, and/or weight, and were excluded from the analyses. At time two, *n* = 434 (76%) of the time one participants answered the survey. Participants reported on their ethnicity through an open-ended written response. Approximately, 40% identified as having an Asian background and an additional 34% of participants described their ethnicity as White. In place of ethnicity, 26% stated their country of origin (e.g., citizen status).

Demographic characteristics (age, sex, and weight and height) were collected, and measures of weight stigmatization, appearance evaluation (i.e., body image), fear of negative appearance evaluation, enjoyment of PA and sport, and frequency of participation in PA or sport in the past 60 days were administered. Additionally, a newly developed measure of the Tendency to Avoid Physical Activity and Sport (TAPAS) because of weight and appearance-related concerns was administered at time one and time two. 

### 2.2. Weight Stigma

To measure weight stigma, we used the modified five-item Perception of Teasing Scale (POTS) [20,21]. The POTS is a two-component measure of experienced weight stigma that assesses frequency and associated perceived harm of weight stigma to the individual. Participants first rate how often they were teased about their weight in the past 12 months using a 5-point Likert scale (1 = ‘never’, and 5 = ‘very often’), and then indicate how upset that teasing made them feel (0 = ‘not upset’, and 5 = ‘very upset). Higher scores indicate greater weight stigma.

### 2.3. Appearance Evaluation

The Multidimensional Body-Self Relations Questionnaire-Appearance Scales [22] is a 34-item measure assessing appearance evaluation, appearance orientation, overweight preoccupation, self-classified weight, and body areas satisfaction. The seven-item appearance evaluation subscale was the only measure included for analysis. The appearance evaluation subscale asks participants to indicate their agreement with statements such as, ‘My body is sexually appealing’, on a scale ranging from 1 = strongly disagree to 5 = strongly agree. Higher scores indicate greater satisfaction with physical appearance.

### 2.4. Fear of Negative Appearance Evaluation

The Fear of Negative Appearance Evaluation Scale (FNAE) [23] is a 6-item scale examining concerns about having one’s body and appearance evaluated by others [24]. Participants are asked to respond to items such as “It bothers me if I know someone is judging my physical shape” using a five-point scale (1 = ‘not at all’, and 5 = ‘extremely)’.

### 2.5. Enjoyment of Physical Activity and Sport

A modified 16 item version of the Physical Activity Enjoyment Scale (Enjoyment) was used to assess enjoyment of PA and sport [25,26]. The original scale only assessed enjoyment of a single PA or sport at that specific point in time (e.g., hockey). Because we were interested in the enjoyment of PA and sport more broadly, we modified the introductory statement of the scale to say the following: “The following scale asks about how you generally feel when you are engaged in physical exercise for fitness and health (e.g., running, playing sport, going to the gym).” This allowed for the assessment of enjoyment of PA and the sport concept more generally. Consistent with the original scale [26], participants indicated their agreement/disagreement to 16 statements (e.g., I enjoy it; It frustrates me; I get something out of it), using a five-point Likert scale (1 = totally disagree to 5 = totally agree).

### 2.6. Participation in Physical Activity or Sport

The present study was interested in whether individuals tend to avoid PA and sport, rather than assess intensity or whether people meet recommended levels (e.g., moderate, vigorous) of PA and sport. Accordingly, and consistent with previous validation studies assessing participation in PA [27,28], we measured PA and sport participation with two single-item questions, i.e., “How many days in the past 2 months did you participate in vigorous (e.g., getting a sweat/puffing) physical activity (e.g., gym workout, jogging, cycling)” and “How many days in the past 2 months did you participate in vigorous sport (e.g., tennis, football etc.)?”. Participants responded by indicating the number of days they participated in sport or PA (possible range 0–60 days). Previous work suggests that self-report of participation PA and sport is appropriate for periods under 90 days prior [29].

### 2.7. Tendency to Avoid Physical Activity and Sport Scale (TAPAS)

There are currently no formal measures of the tendency to avoid PA and sport because of weight or appearance-related concerns. A literature search identified only eight studies that had examined, albeit in an ad hoc manner, the avoidance of physical activity (exercise) and/or sport due to either weight or physical appearance-related concerns [8,26,30,31,32,33,34,35]. None of these studies used more than one or two items to assess the tendency to avoid PA and/or sport due to weight, stigma or appearance-related concerns. The psychometric properties of these one or two item measures were also absent. Accordingly, we sought to develop a new scale for assessing the tendency of avoidance of physical activity and sport because of body related concerns, the TAPAS construct. We followed traditional scale development guidelines [36,37,38], and drew on the COSMIN checklist (Consensus-based Standards for the selection of health status Measurement Instruments) [39] throughout the construction of the scale. As a first step we conducted a brief mixed-methods survey with open ended qualitative response options asking participants (*n* = 496) to describe their reasons for avoiding participation in physical activity and sport. Four-hundred and ninety-three participants provided a text response of varying detail to both of the avoidance questions (smallest, one word, longest, 133 words). A thematic analysis of the survey responses by two researchers generated 11 broad themes identified for not participating in either PA or sport (e.g., injury, time, accessibility, and cost). The present study was interested in three identified themes that centred on weight and physical appearance-related concerns e.g., “worried people will tease me due to my weight”, “I am uncomfortable and worried about my physical appearance in front of other people” and “embarrassment that I will be unable to do it, or look stupid doing it”.

Three subject matter experts (including KO and JL) used the participant responses to develop an initial pool of 21 items (questions) assessing the tendency to avoid PA and sport due to weight and physical appearance-related concerns. We then assessed whether items were worded simply with terminology common to all English-speaking age groups, and avoided a double barreled structure [36]. The items were then presented to nine university students to assess interpretability and improve face validity. We subsequently modified three items and removed six items because of issues with face validity and interpretability. An additional five items were removed because of redundancy (similar wording). The remaining 10 questions were then written to be relevant to the context of both PA and sport participation. This resulted in 10 items assessing the tendency to avoid PA and 10 items assessing the tendency to avoid sport. For example, the question “I find myself avoiding participating in sport because of my weight” was repeated under a separate heading, with the term ‘sport’ replaced by ‘physical activity’. Participants indicated their agreement or disagreement using a 5-point Likert scale anchored with 1 = strongly disagree and 5 = strongly agree. We conducted statistical analyses including exploratory factor analysis, internal reliability, and construct and predictive validity tests.

### 2.8. Analysis

Because of known differences between men and women on the measures of interest (e.g., sport participation, body image, weight stigma), sex differences between men and women were examined using ANOVA. Pearson’s correlation coefficients were used to look at simple bivariate relationships between all measures. Hierarchal multivariate linear regressions were used to test the TAPAS with established measures and participation in PA or sport and the enjoyment of PA and sport. Principal component analysis with direct oblimin rotation was used to assess the factor structure of the 20 items assessing the tendency to avoid PA and sport. We used the direct oblimin rotation over the Varimax rotation because there were theoretical grounds for the correlations of interest, and Direct Oblimin adjusts for the possibility that the components are intercorrelated [40]. All statistical analyses were performed using SPSS software, version 28, IBM, Chicago, IL, USA.

## 3. Results

Internal reliability and factor analysis were conducted on the established scales which were used in this study. The weight stigma frequency and upset component scales were highly correlated with one another (*r* = 0.84). Consistent with previous research [5,21], a single weight stigma score was calculated by combining the weight stigma-frequency (and upset components) and dividing by two. Factor analysis showed a strong single factor solution (Eigen value = 6.84) for the measure, and Cronbach’s alpha for the measure in this sample was good (0.93). The total weight stigma scores could range from 2.5 to 25. Cronbach’s alpha for appearance evaluation and fear of negative appearance evaluation for this study were both good (α = 0.85; α = 0.94, respectively). A confirmatory factor analysis showed that the modified PACES scale retained a single factor solution for enjoyment (eigen value = 8.87). Similarly, the scale internal consistency was good (α = 0.94). A mean item score (1–5) is calculated with higher mean scale scores, indicating greater enjoyment when participating in PA and sport.

Sex differences (means and standard deviations) for all variables of interest are displayed in Table 1. Women reported significantly higher TAPAS and FNAE scores than men. Women also reported significantly lower appearance evaluation (greater appearance dissatisfaction), lower participation of PA and sport, and lower enjoyment of PA and sport, than men.

### 3.1. TAPAS Scale Refinement

Cronbach’s alpha was 0.98 for the full 20 items, indicating good internal consistency. We expected a two-factor solution; however, the factor analysis (principal component analysis) suggested a single factor solution (Eigenvalue of 7.39), with the next closest factor (Eigenvalue of 0.64) well below Kaiser’s criterion of 1. Item factor loadings also suggested that the ten items assessing PA avoidance and ten items assessing sport avoidance, respectively, were nearly identical. This suggests that the items were capturing the same construct regardless of the different exercise context (i.e., PA vs. sport). Accordingly, we reduced the overall scale to 10 items by selecting the five items from the PA context and five items from the sport context with the highest factor loadings (see Table 2).

We re-ran the principal component analysis with the final ten items. Again, a single factor solution was found (Eigenvalue of 7.40), explaining 74% of the variance, with the next closest factor below Kaiser’s criterion of 1 (0.72). Sampling adequacy for the analysis was good (Kaiser–Meyer–Olkin = 0.94), with Bartlett’s Test of Sphericity significant (*p* = 0.0005) indicating the suitability of the sample for principal component analysis. Table 2 displays the resulting scale items, factor loadings and means and standard deviations for men and women. Cronbach’s alpha for the ten-item scale remained good (α = 0.96) and was similar for both men (α = 0.95) and women (α = 0.96).

### 3.2. Predictive Validity

Table 3 displays the bivariate relationships between the TAPAS and other measures of interest. The pattern of relationships was similar for women and men. To establish the predictive qualities of the TAPAS, we ran three hierarchical regression analyses to test associations with the three primary outcomes of interest (PA participation, sport participation, enjoyment of PA and sport). In the first step involving the models, we entered age, sex and BMI. In the second step, we entered weight stigma, appearance evaluation, and FNAE. TAPAS was entered in the third step of the models. All models were significant, with sex significant across all models.

The full model accounted for 18% (*p* < 0.001) of the variance in reported PA participation. Appearance evaluation (*B* = 2.80, *SE* = 0.99, *β* = 0.14, *t* = 2.83, *p* < 0.005), FNAE (*B* = 0.33, *SE* = 0.12, *β* = 0.14, *t* = 2.85, *p* < 0.005), and TAPAS (*B* = −0.40, *SE* = 0.08, *β* = −0.27, *t* = −5.35, *p* < 0.001), were the only significant variables in the final model. The entry of the TAPAS in the final step of the model accounted for an additional 4.3% of the variance in PA participation over and above all other variables in the model.

For sport participation, the full model accounted for 14% (*p* < 0.001) of the variance. The entry of TAPAS in the final step accounted for an additional 3.5% of unique variance, with both weight stigma (*B* = 0.27, *SE* = 0.12, *β* = 0.10, *t* = 2.18, *p* < 0.030), and TAPAS (*B* = −0.29, *SE* = 0.06, *β* = −0.24, *t* = −4.67, *p* < 0.001) significantly associated with sport participation in the final model. That is, those who experience greater weight stigmatization and report a greater tendency to want to avoid PA and sport are less likely to participate in sport.

For enjoyment of PA and sport, the final model accounted for 22% (*p* < 0.001) of the variance. In the final model, the entry of TAPAS in the final step of the model accounted for an additional 7.1% of variance in the enjoyment of PA and sport. Only appearance evaluation (*B* = 0.23, *SE* = 0.05, *β* = 0.23, *t* = 4.58, *p* < 0.001) and the TAPAS (*B* = −0.03, *SE* = 0.00, *β* = −0.35, *t* = −6.89, *p* < 0.001) were significantly related to the enjoyment of PA and sport. That is, greater TAPAS scores and lower body satisfaction were associated with lower enjoyment of PA and sport.

### 3.3. Test-Retest Reliability

Four hundred and thirty-four (76%) of the participants who completed the first survey also completed the second survey after two weeks. Cronbach’s alpha for the first data sets was 0.96 at both time points. Two-week test-retest reliability for the TAPAS was *r* = 0.82 (*p* < 0.0005). In the first administration, the mean scale score was 19.62 (SD = 10.14), and for the second administration the mean scale score was 20.29 (SD = 10.31). This small difference was not significantly different between time points (*p* > 0.05).

## 4. Discussion

Although research has examined the structural and environmental factors affecting PA and sport participation, there has been little research on the psychosocial reasons for avoiding participation in PA and sport. This is in part due to a lack of psychometrically sound measures to assess the psychosocial reasons for avoiding PA and sport. We developed and tested a new measure that assesses the tendency to want to avoid PA and sport because of psychological concerns about weight, weight stigma, and physical appearance. The TAPAS was found to have good psychometric properties, with predictive validity across men and women, and good test-retest reliability. The TAPAS predicted unique variance in self-reported participation in PA and sport. Additionally, TAPAS scores were associated with the self-reported enjoyment of PA and sport. Finally, experiences of weight stigma, self-appearance evaluation (body image), and fear of negative appearance evaluations from others were all found to be correlated with TAPAS scores.

The results indicate that psychosocial factors such as weight stigmatization and appearance-related concerns play a central role in the tendency for people to want avoid PA and sport. Notably, women are found to be more likely to avoid PA and sport because of weight and appearance-related concerns than men. Women also tended to avoid PA and sport more, and enjoy PA and sport less, than men. These findings are consistent with the body image and eating disorder literature showing that young women are more affected by weight and appearance-related concerns [21,41], and participate less in PA and sport [15]. Our findings suggest that research on the reasons for non- or low participation in PA and sport would benefit from the further examination of psychosocial factors such as weight stigma and physical appearance related concerns.

Previous work seeking to understand the reasons for decreasing rates of participation in PA and sport consistently suggest that structural and environmental factors (e.g., accessibility, cost) are associated with lower participation [3], yet attempts to address these factors have not resulted in increased participation rates. For example, following the 2012 London Summer Olympics, participation in PA and sport in the UK did not increase despite significant investment and improvements in sporting infrastructure, access and funding [42]. Our findings suggest that in addition to structural and environmental factors, weight stigma and appearance-related concerns may also need to be addressed in order to increase participation in PA and sport [43]. Previous research suggests that private settings may be more suitable than public settings for people who have weight and appearance-related concerns; private settings may also be particularly important for people with social anxiety [44].

Public health and sport policy makers may need to consider psychosocial barriers in developing interventions to address the declining participation levels in PA and sport. Those developing and delivering PA and sport programs need to consider how they may better provide social and physical environments that reduce the chance of weight stigmatization and concerns about physical appearance more broadly. In addition to addressing the social setting of PA and sport, individual-level psychological approaches could also help to reduce the internalized weight stigma and body image concerns that appear to underpin the tendency to avoid PA and sport. For example, Acceptance Commitment Therapy has shown some promise in reducing the impact of weight stigma on body image and weight stigma internalization [45], and may demonstrate utility within the PA and sport context.

There are some limitations to the present study. The present sample was a relatively homogenous group of young people, and the psychosocial factors examined here may not be as pronounced in older populations (>40 years). For example, it is well understood that body image and weight-related concerns decrease with age [46]. Additionally, participation in PA and sport decreases with age due to injuries and other life priorities and responsibilities [47]. Accordingly, the TAPAS and associated measures of interest need to be tested in older populations. The cross-sectional design of the study prohibits causal inferences from being made about the relationships between the psychosocial constructs examined and the tendency to avoid PA and sport. Longitudinal research is needed to understand these potential causal underpinnings.

## 5. Conclusions

Notwithstanding the limitations, the present study provides a new measure for assessing psychosocial factors underpinning the participation in PA and sport. The results show that various appearance and body-related psychosocial factors play an important role in people’s choice to participate in PA and sport. Research needs to examine whether these psychosocial constructs play as important a role with regard to participation in PA and sport as do environmental and economic factors. The present research, along with other studies examining the psychosocial contribution to PA and sport avoidance and participation, suggest that there is a need for more research in this area. The TAPAS is one tool that may help researchers examine this area in more detail in the future.

## Figures and Tables

**Table 1:** Mean (SD) participant ratings for men and women on each of the variables along with F-values, significance levels, and effect sizes for group differences.

Variables	Men (*n* = 151)	Women (*n* = 427)	Total	*F* Value	*p* Value	Effect Size ^†^ *d’*
Age	20.21 (1.92)	19.70 (2.20)	19.83 (2.14)	6.45	0.011	0.26
BMI	23.22 (3.20)	22.22 (3.49)	22.48 (3.45)	9.60	0.002	0.30
Weight stigma	6.00 (4.29)	6.49 (4.83)	6.36 (4.70)	1.19	0.276	0.11
Appearance Evaluation	3.49 (0.76)	3.17 (0.76)	3.25 (0.77)	19.00	0.000	0.24
FNAE	16.23 (5.70)	19.00 (6.37)	18.26 (6.32)	22.06	0.000	0.45
TAPAS	16.16 (8.69)	20.86 (10.40)	19.63 (10.18)	24.24	0.000	0.49
PA Participation	22.40 (16.98)	12.26 (13.28)	14.91 (15.0)	55.88	0.000	0.67
Sport Participation	15.21 (14.87)	6.78 (10.35)	8.98 (12.25)	58.02	0.000	0.67
Enjoyment	4.11 (0.66)	3.72 (0.77)	3.82 (0.76)	28.51	0.000	0.55

Note: ^†^ Cohen’s *d’*, small effect ≈ 0.2 moderate effect ≈ 0.5, large effect ≈ 0.8+. Weight stigma = Perception of teasing scale, Appearance evaluation = Self-appearance evaluation, FNAE = Fear of negative appearance evaluation, TAPAS = Tendency to avoid physical activity and sport, PA Participation = Physical activity participation, Sport Participation = Participation in sport, Enjoyment = The physical activity enjoyment scale.

**Table 2:** Scale items and factor loadings of TAPAS.

Scale Item	Men	Women		
	Mean (SD)	Mean (SD)	Mean (SD)	Loadings
I find myself avoiding participating in sport because of my weight	1.46 (0.91)	1.74 (1.03)	1.67 (1.01)	0.810
I avoid participating in sport because of my fear of being judged about my lack of physical ability	1.65 (1.07)	2.39 (1.37)	2.20 (1.34)	0.801
I worry about participating in sport because I don’t like how my body looks when playing sport	1.54 (0.96)	2.02 (1.21)	1.90 (1.17)	0.901
I am afraid other people will notice my physical flaws when I participate in sport	1.68 (1.07)	2.14 (1.25)	2.03 (1.23)	0.884
I am concerned about what other people think of my appearance when I participate in sport	1.76 (1.20)	2.27 (1.28)	2.14 (1.28)	0.873
I avoid physical activity because I might get teased about my weight	1.36 (0.84)	1.62 (0.98)	1.56 (0.95)	0.847
I avoid physical activity because of my fear of being judged about my physical appearance	1.50 (0.93)	1.93 (1.20)	1.83 (1.15)	0.932
I avoid physical activity because I worry that people may make negative comments about my body	1.49 (0.92)	1.83 (1.14)	1.75 (1.10)	0.907
I avoid physical activity because I worry people may be thinking negatively about my physical appearance	1.52 (0.96)	1.96 (1.21)	1.85 (1.16)	0.926
I would prefer to participate in physical activity in a more private setting	2.15 (1.33)	2.90 (1.48)	2.70 (1.48)	0.697

Note: Items are rated on a 5-point scale from 1 = strongly disagree to 5 = strongly agree.

**Table 3:** Pearson’s product moment correlations between TAPAS and measures of weight stigma and appearance concerns for men and women.

	Men	Women	Total
1. Age	−0.14	−0.04	−0.08
2. BMI	0.14	0.26 ***	0.20 ***
3. Weight stigma	0.46 ***	0.50 ***	0.49 ***
4. Appearance evaluation	−0.51 ***	−0.47 ***	−0.50 ***
5. FNAE	0.35 ***	0.50 ***	0.50 ***
6. PA Participation	−0.22 **	−0.22 ***	−0.26 ***
7. Sport participation	−0.16	−0.17 ***	−0.21 ***
8. Enjoyment	−0.50 ***	−0.33 ***	−0.39 ***

** correlations significant at *p* < 0.01 level; *** correlations significant at *p* < 0.001.

## Data Availability

Data from the present study can be requested from the corresponding author Kerry O’Brien (kerrykez@gmail.com).
